# Waist-corrected BMI predicts incident diabetes mellitus in a population-based observational cohort study

**DOI:** 10.3389/fendo.2023.1186702

**Published:** 2023-06-09

**Authors:** Nana Wang, Yuying Li, Chuanji Guo

**Affiliations:** ^1^ Endocrinology Department, Shengjing Hospital of China Medical University, Shenyang, China; ^2^ Health Commission of Tacheng Area, Tacheng, China; ^3^ Department of Hospital Administration Office, Shengjing Hospital of China Medical University, Shenyang, China

**Keywords:** diabetes mellitus, waist-corrected body mass index, body mass index, waist circumference, waist-to-height ratio

## Abstract

**Introduction:**

Waist-corrected body mass index (wBMI), which combines BMI and waist circumference (WC) measurements, has proven superior to either measure alone for predicting obesity but has not yet been applied to the prediction of diabetes mellitus (DM).

**Methods:**

Over a 5-year period, 305,499 subjects were eligible for this study based on citizen health check-ups in the Tacheng Area of northwest China. Diagnosis of DM was defined as the end point.

**Results:**

After exclusion, a total of 111,851 subjects were included in the training cohort and 47,906 in the validation cohort. Participants of both sexes with wBMI in the upper quartiles had significantly higher incidence of DM than those with wBMI in the lower quartiles (log-rank χ^2 = ^236, p< 0.001 for men; log-rank χ^2 = ^304, p< 0.001 for women). After adjusting for multiple variables, WC, BMI, wBMI, and waist-to-height ratio (WHtR) were all independent predictors for diabetes. In men, the adjusted hazard ratios (HRs) of wBMI for diabetes for the second, third, and fourth quartiles were 1.297 [95% CI: 1.157, 1.455], 1.664 [95% CI: 1.493, 1.853], and 2.132 [95% CI: 1.921, 2.366], respectively, when compared with the first quartile. In women, they were 1.357 [95% CI: 1.191, 1.546], 1.715 [95% CI: 1.517, 1.939], and 2.262 [95% CI: 2.010, 2.545], respectively. Compared with WC, BMI, and WHtR, wBMI had the highest C-index in both men (0.679, 95% CI: 0.670, 0.688) and women (0.730, 95% CI: 0.722, 0.739). Finally, a nomogram was constructed to predict incident DM based on wBMI and other variables. In conclusion, wBMI had the strongest predictive capacity for incident DM when compared with WC, BMI, and WHtR, especially in women.

**Discussion:**

This study provides a reference for advanced investigation of wBMI on DM and other metabolic diseases in the future.

## 1 Introduction

Overweight and obesity are well-known risk factors for incident type 2 diabetes mellitus (T2DM). Body mass index (BMI) is the most common index for assessing overall adiposity because it is measured easily and is strongly associated with total body fat mass. High BMI has been associated with increased risk of T2DM (1). In study of Pima Indians, the age-adjusted risk ratio for developing diabetes was 90.3 for individuals with BMI ≥40 kg/m^2^ compared to those with BMI<20 kg/m^2^ (2). In a study of female nurses, the risk for incident diabetes increased 93.2-fold in individuals whose BMI increased from<22 kg/m^2^ at age 18 to ≥35.0 kg/m^2^ at age 30–55, compared to individuals who maintained a steady weight (3). BMI has long been a traditional, routine, and important indicator to be monitored in patients with obesity or hyperglycemia.

However, recent studies have demonstrated the limitations of BMI. It evaluates general obesity but does not account for body fat distribution (4). While some studies have associated BMI with abdominal obesity, others show divergence between the two measures, suggesting that BMI may not accurately reflect the distribution of fat in the body (5). Individuals who have abdominal obesity but are lean according to BMI show increased prevalence of cardiovascular disease and diabetes (6). Other studies have shown that a larger waist circumference (WC) increases the future risk of cardiovascular disease and diabetes by two- to threefold for a given BMI (7, 8).

Because of the aforementioned shortfalls of BMI in evaluating adipose distribution, other indexes have been adopted to assess body shape, including WC, WaisttoHip Ratio, WaisttoHeight Ratio (WHtR), Body Adiposity Index, A Body Shape Index, and Visceral adiposity Index (9–16). Recently, waist-corrected BMI (wBMI), a new and simple indicator combining BMI and WC, was developed by Antonini-Canterin et al. in 2018 to evaluate overfat and obese patients. It has the advantage of considering global fat mass in conjunction with fat distribution and therefore could overcome the limitations of BMI or WC alone. It is calculated by the equation below (17), in which body weight (BW) is measured in kg, WC in m, and height (H) in m.


$$wBMI=\frac{BW*WC}{H^{2}}$$

Studies of wBMI demonstrated that it outperformed BMI, WC, and WHtR most dramatically in predicting adverse cardiac remodeling patterns, increased arterial stiffness, increased insulin resistance, and unfavorable lipid profile (17). Moltrer et al. evaluated the accuracy of wBMI for classifying overfat and obese patients identified by fat mass percentage in comparison to BMI, WC, and WHtR. They found that wBMI had the greatest discriminating capacity for female patients. wBMI is therefore an accurate indicator for healthcare professionals to identify overfat and obese patients and monitor them during the course of treatment (18).

However, the predictive effect of wBMI on diabetes has not been studied. Considering the advantages offered by wBMI in identifying overfat and obese patients, this study was designed to compare the predictive capacity of wBMI with BMI, WC, and WHtR (12, 19). The present study compared concordance indexes (C-index) of Cox regression analysis across body composition measures and constructed a predictive nomogram including wBMI and other important variables.

## 2 Materials and methods

### 2.1 Data source

Tacheng Area is a region of Xinjiang Province in northwest China with a population of 1.1 million. All citizens in this area received free yearly health checkups beginning in 2016 as part of a social welfare program encompassing 608 health checkup organizations in seven cities. The present study analyzed health checkup data from 1 January 2016 to 31 December 2020. During this time, 305,499 adult individuals (18–117 years old) received annual checkups.

The study was approved by the Ethical Review Committee of Shengjing Hospital of China Medical University (No. 2021PS633K). Informed consent was waived due to the non-interventional study design. The study was conducted in accordance with guidelines set forth in the Declaration of Helsinki.

### 2.2 Study design

Participants aged more than 18 years old were enrolled, their age, sex, height, weight, and WC were recorded at every visit. Fasting plasma glucose (FPG), serum lipids, and liver and kidney function were also examined. Information on diabetes family history, alcohol consumption, previous disease history, current disease status, present medication, and habits of smoking, drinking, and exercise were collected in self-reported questionnaires at the first visit in 2016.

Subjects were excluded if their baseline age was<18 years; they had a history of diabetes, malignancy, severe liver or kidney dysfunction, or hyperthyroidism or other endocrine disease that affects blood glucose; they took medication that affects glucose levels (e.g., glucocorticoids, antidepressants); they had fewer than three visits or were missing data; and if they were pregnant.

### 2.3 Definitions of variables

There were four means of classifying patients as diabetic: self-reported, FPG ≥7.0 mmol/L, HbA1c ≥6.5%, or use of diabetic medications (including special diet, weight control medication, oral medication, insulin injection, or intake of Chinese traditional medicine).

Tobacco smoking was classified as “never” (fewer than 100 cigarettes smoked in lifetime), “ever” (smoked at least 100 cigarettes in their lifetime but has quit smoking for at least the previous 12 months), or “current smoker” (including daily smokers and non-daily or occasional smokers). Alcohol history was classified as “never,” “mild drinker” (<30 g alcohol/day), or “heavy drinker” (≥30 g alcohol/day). Physical exercise was defined as more than 30 min exercise at a time, and the frequency of physical exercise was classified as “seldom” (<1 time/week), “occasionally” (1–3 times/week), and “frequently” (>3 times/week). Diet pattern was self-reported as either “Mediterranean” (predominantly vegetables, fruits, low-fat dairy, and legumes), “meat” (predominantly red and processed meat products), or “balanced.” Height, weight, and WC were measured according to standard methods. BMI was calculated as weight in kilograms divided by height (in meters) squared. WHtR was calculated as WC in meters divided by height in meters.

### 2.4 Statistical analysis

Continuous data are presented as mean (SD) and categorical variables as frequencies. Continuous variables were compared between two groups using an independent samples *t*-test after Leneve’s test for equality of variance. χ^2^ test was used to compare categorical variables. Predictive analysis of wBMI, BMI, WC, and WHtR for incident DM was analyzed by Cox regression after adjusting for confounding variables. Predictive abilities of the various body composition measures were compared by C-indexes. A nomogram was developed using weighted estimators corresponding to each covariate derived from fitted Cox regression coefficients and estimates of variance. Validation of the nomogram was assessed by a calibration curve. A calibration plot was generated to compare the actual Kaplan–Meier survival estimates with predicted survival probabilities. Cox regression and the nomogram were calculated in R software (version 4.0.3) with survival (version 3.4-0), rsm (version 2.10.3), survcomp (1.48.0), and survminer (version 0.4.9) packages. Statistical significance was set at *p<* 0.05.

## 3 Results

### 3.1 Baseline characteristics

Individuals were excluded for missing wBMI information (n = 14,814); having diabetes at baseline (n = 36,532); being younger than 18 years old (n = 40,137); missing data for FPG, serum lipids, or liver and kidney function (n = 42,050); having fewer than three visits (n = 18,601); and other reasons (n = 3,608). After exclusion, 159,757 adult subjects were included in this study. Subjects were randomly divided into training and validation cohorts by 70:30 ratio to yield 111,851 training subjects and 47,906 validation subjects. There were 52,758 men and 59,093 women, the mean age was 44.9 ± 14.0 years, and the mean follow-up time was 3.3 ± 0.7 years (range, 0.3–4.9 years) in the training cohort. There were 22,630 men and 25,276 women, the mean age was 45.8 ± 13.6 years, and the mean follow-up time was 3.4 ± 0.7 years (range, 0.2–5.0 years) in the validation cohort). **Supplementary Figure S1** shows the participant selection process. **Supplementary Table S1** presents the baseline characteristics of subjects in validation cohort.

Because wBMI differs between men and women (18), participants were divided by sex. Compared to non-diabetic patients, at baseline, DM patients of both sexes were older (52.1 ± 12.8 years for men, 55.2 ± 12.4 years for women); had higher systolic blood pressure (127.8 ± 10.5 mmHg for men and 122.8 ± 12.0 mmHg for women) and FPG (5.48 ± 0.84 mmol/L for men and 5.51 ± 0.82 mmol/L for women); larger WC (93.1 ± 12.6 cm for men, 88.2 ± 12.2 cm for women), BMI (27.0 ± 4.0 kg/m^2^ for men, 26.6 ± 4.4 kg/m^2^ for women), wBMI (25.4 ± 6.9 kg/m for men, 23.7 ± 7.1 kg/m for women), and WHtR (0.59 ± 0.53 for men, 0.55 ± 0.09 for women); higher percentage of DM family history (4.3% for men, 4.1% for women) and history of high blood pressure (HBP) (20.9% for men, 21.7% for women); lower education level (12.9% of men and 10.6% of women received >9 years of education); and less physical exercise (6.8% of men and 6.1% of women were frequently active) (**Table 1**).

**Table 1:** Baseline characteristics of participant by incident diabetes mellitus in men and women.

	Male			Female		
	DM	NDM	p	DM	NDM	p
n	3,657	49,101		3,272	55,821	
Age (years)	52.1 (12.8)	45.2 (13.9)	<0.001	55.2 (12.4)	45.2 (13.2)	<0.001
Pulse (rpm)	76.4 (18.6)	75.5 (25.1)	0.035	77.5 (21.4)	76.4 (26.5)	0.030
SBP (mmHg)	127.8 (10.5)	125.4 (10.3)	<0.001	122.8 (12.0)	121.5 (11.7)	0.002
DBP (mmHg)	80.4 (9.5)	78.4 (8.2)	<0.001	75.8 (9.3)	75.5 (8.4)	0.331
WC (cm)	93.1 (12.6)	88.8 (12.0)	<0.001	88.2 (12.2)	82.8 (12.0)	<0.001
BMI (kg/m^2^)	27.0 (4.0)	25.5 (3.8)	<0.001	26.6 (4.4)	24.6 (4.1)	<0.001
wBMI (kg/m^2^*m)	25.4 (6.9)	22.9 (6.0)	<0.001	23.7 (7.1)	20.7 (6.0)	<0.001
WHtR	0.59 (0.53)	0.53 (0.48)	0.01	0.55 (0.09)	0.52 (0.50)	<0.001
DM family history (Yes, % (n))	4.4 (160)	2.0 (1,001)	<0.001	4.1 (135)	2.1 (1,169)	<0.001
Urban (yes, % (n))	37.8 (1,382)	39.1 (19,201)	0.106	38.6 (1,263)	39.2 (21,910)	0.459
>9-year education (Yes, % (n))	12.9 (472)	14.8 (7,286)	0.001	10.6 (344)	15.2 (8,467)	<0.001
Exercise (% (n))			<0.001			<0.001
Seldom	82.8 (3,028)	84.6 (41,560)		83.2 (2,725)	85.4 (47,683)	
Occasionally	10.4 (380)	4.7 (2,300)		10.5 (347)	3.9 (2,200)	
Frequently	6.8 (249)	10.7 (5,241)		6.1 (200)	10.6 (5,938)	
Diet (% (n))			0.015			0.150
Mediterranean	2.0 (73)	2.3 (1,124)		3.1 (101)	3.3 (1,839)	
Balance	95.4 (3,490)	94.3 (46,319)		95.0 (3,107)	94.2 (52,609)	
Meat	2.6 (94)	3.4 (1,658)		2.0 (64)	2.5 (1,373)	
Smoker (% (n))			0.004			0.049
Never	56.3 (2,058)	57.2 (28,105)		98.6 (3,227)	99.0 (55,279)	
Ever	5.4 (197)	4.2 (2,076)		0.09 (3)	0.1 (66)	
Present	38.3 (1,402)	38.6 (18,920)		1.3 (42)	0.9 (476)	
Drinker (% (n))			0.005			0.021
Never	60.5 (2,212)	62.2 (30,534)		96.5 (3,158)	95.8 (53,469)	0.370
Mild	34.2 (1,252)	33.6 (16,491)		3.2 (105)	4.0 (2,259)	0.487
Heavy	5.3 (193)	4.2 (2,076)		0.3 (9)	0.2 (93)	Ref
HBP (Yes, % (n))	20.9 (763)	5.6 (2734)	<0.001	21.6 (707)	4.1 (2,310)	0.004
CHD (Yes, % (n))	0.1 (4)	0.08 (41)	0.619	0.1 (4)	0.1 (57)	0.734
Cerebral stroke (Yes, % (n))	0.3 (10)	0.1 (58)	0.026	0.3 (11)	0.2 (105)	0.063
FPG (mmol/L)	5.48 (0.84)	4.97 (0.71)	<0.001	5.51 (0.82)	4.92 (0.68)	<0.001
ALT (U/L)	29.5 (21.7)	27.3 (18.6)	<0.001	26.6 (17.4)	20.8 (14.4)	<0.001
AST (U/L)	24.5 (11.4)	23.8 (11.7)	<0.001	23.1 (12.8)	21.7 (10.7)	<0.001
SCr (μmol/L)	78.1 (23.9)	77.3 (21.6)	0.011	69.0 (22.8)	66.2 (21.9)	<0.001
TC (mmol/L)	4.78 (1.25)	4.66 (1.21)	<0.001	4.83 (1.30)	4.58 (1.20)	<0.001
TG (mmol/L)	1.81 (1.35)	1.49 (1.10)	<0.001	1.64 (1.12)	1.25 (0.90)	<0.001
LDL (mmol/L)	2.78 (1.02)	2.70 (0.98)	<0.001	2.77 (1.04)	2.60 (0.96)	<0.001
HDL (mmol/L)	1.43 (0.57)	1.46 (0.59)	0.008	1.50 (0.59)	1.54 (0.58)	<0.001

SBP, systolic blood pressure; DBP, diastolic blood pressure; WC, waist circumstance; BMI, body mass index; wBMI, waistcorrected BMI; WHtR, WaisttoHeight Ratio; DM, diabetes mellitus; HBP, hypertension; CHD, coronary heart disease; FPG, fast plasma glucose; ALT, alanine aminotransferase; AST, aspartate aminotransferase; SCr, serum creatinine; TC, total cholesterol; TG, triglycerides; LDL, low-density lipoprotein; HDL, high-density lipoprotein.

### 3.2 wBMI is a risk factor of incident DM

**Figure 1:**
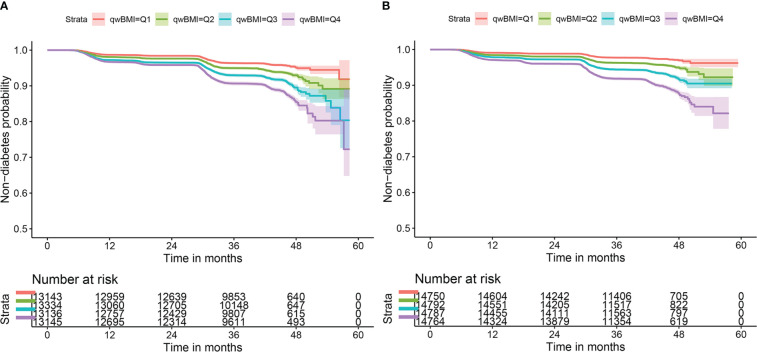
Kaplan–Meier survival curves of non-diabetes by wBMI quartiles in both sexes. Incident diabetes mellitus risk increases with wBMI quartiles in **(A)** men and **(B)** women. Log-rank χ^2 = ^236, p< 0.001 for men; log-rank χ^2 = ^304, p< 0.001 for women. wBMI, waist-corrected body mass index.

Unadjusted 2- and 4-year DM incidence in this cohort was 2.9% and 6.9%, respectively, in men 2.5% and 5.5% in women. As shown in **Figure 1**, participants of both sexes with wBMI in the upper quartiles had significantly greater DM incidence than those with wBMI in the lower quartiles (log-rank χ^2 = ^236, p< 0.001 for men; log-rank χ^2 = ^304, p< 0.001 for women).

Univariate Cox regression models showed that wBMI was a significant predictor of incident DM (men: Q2: HR 1.484 [95% CI: 1.326, 1.662]; Q3: HR 2.080 [95% CI: 1.870, 2.315]; Q4: HR 2.829 [95% CI: 2.554, 3.134]; women: Q2: HR 1.532 [95% CI: 1.294, 1.814]; Q3: HR 2.371 [95% CI: 2.027, 2.773]; Q4: HR 3.196 [95% CI: 2.749, 3.717]). The HRs increased with elevated wBMI quartiles for both sexes (**Table 2**). Age, DM family history, FPG, education years, current habits of smoking and exercise, and diagnosis of HBP and cerebral stroke were statistically significant for incident DM and were used to adjust for incident DM in the following multivariable Cox regression models.

**Table 2:** Hazard ratios of WC, BMI, wBMI and WHtR by incident DM in men and women.

	Male				Female			
	Model 1		Model 2		Model 1		Model 2	
	HR (95%CI)	p	HR (95%CI)	p	HR (95%CI)	p	HR (95%CI)	p
BMI						<0.001		<0.001
Q1	Ref	<0.001	Ref		Ref		Ref	
Q2	1.379 (1.239, 1.535)	<0.001	1.249 (1.122, 1.390)	<0.001	1.684 (1.484,1.911)	<0.001	1.382 (1.217, 1.568)	<0.001
Q3	1.824 (1.646, 2.022)	<0.001	1.521 (1.371, 1.686)	<0.001	2.453 (2.177,2.765)	<0.001	1.679 (1.489, 1.894)	<0.001
Q4	2.477 (2.248, 2.729)	<0.001	1.864 (1.690, 2.056)	<0.001	3.626 (3.236,4.064)	<0.001	2.189 (1.950, 2.457)	<0.001
WC		<0.001		<0.001		<0.001		<0.001
Q1	Ref		Ref		Ref		Ref	
Q2	1.479 (1.323, 1.652)	<0.001	1.329 (1.189, 1.4856)	<0.001	1.566 (1.384,1.772)	<0.001	1.323 (1.169,1.498)	<0.001
Q3	1.905 (1.713, 2.119)	<0.001	1.579 (1.419, 1.757)	<0.001	2.335 (2.084,2.617)	<0.001	1.657 (1.478,1.859)	<0.001
Q4	2.756 (2.491, 3.049)	<0.001	2.184 (1.972, 2.420)	<0.001	3.463 (3.099,3.868)	<0.001	1.968 (1.757,2.204)	<0.001
wBMI		<0.001				<0.001		<0.001
Q1	Ref		Ref		Ref		Ref	
Q2	1.484 (1.326, 1.662)	<0.001	1.297 (1.157, 1.455)	<0.001	1.684 (1.484,1.911)	<0.001	1.357 (1.191, 1.546)	<0.001
Q3	2.080 (1.870, 2.315)	<0.001	1.664 (1.493, 1.853)	<0.001	2.453 (2.177,2.765)	<0.001	1.715 (1.517, 1.939)	<0.001
Q4	2.829 (2.554, 3.134)	<0.001	2.132 (1.921, 2.366)	<0.001	3.626 (3.236,4.064)	<0.001	2.262 (2.010, 2.545)	<0.001
WHtR		<0.001				<0.001		<0.001
Q1	Ref		Ref		Ref		Ref	
Q2	1.447 (1.300, 1.611)	<0.001	1.296 (1.163, 1.443)	0.037	1.566 (1.384,1.772)	<0.001	1.372 (1.217,1.547)	<0.001
Q3	1.969 (1.784, 2.173)	<0.001	1.554 (1.407, 1.716)	<0.001	2.335 (2.084,2.617)	<0.001	1.675 (1.487, 1.887)	<0.001
Q4	2.743 (2.488, 3.025)	<0.001	1.889 (1.710, 2.086)	<0.001	3.463 (3.099,3.868)	<0.001	1.947 (1.741, 2.178)	<0.001

Model 1, unadjusted for variables.

Model 2, adjusted for age, DM family history, FPG, education years, current habit of smoking and exercise, with diagnosis of HBP and cerebral stroke.

WC in men: Q1: ≤81.0; Q2: 81.1–89.0; Q3: 89.1–96.0; Q4: ≥96.1; in women: Q1: ≤75.0; Q2: 75.1–82.0; Q3: 82.1–90.0; Q4: ≥90.1.

BMI in men: Q1: ≤22.9; Q2: 23.0–25.3; Q3: 25.4–27.8; Q4: ≥27.9; in women: Q1: ≤21.9; Q2: 22.0–24.2; Q3: 24.3–27.0; Q4: ≥27.1.

wBMI in men: Q1: ≤18.8; Q2: 18.9–22.3; Q3: 22.4–26.4; Q4: ≥26.5; in women: Q1: ≤16.7; Q2: 16.8–19.9; Q3: 20.0–23.9; Q4: ≥24.0.

WHtR in men: Q1: ≤0.48; Q2: 0.49–0.52; Q3: 0.53–0.57; Q4: ≥0.58; in women: Q1: ≤0.47; Q2: 0.48–0.52; Q3: 0.53–0.56; Q4: ≥0.57.

**Figure 2:**
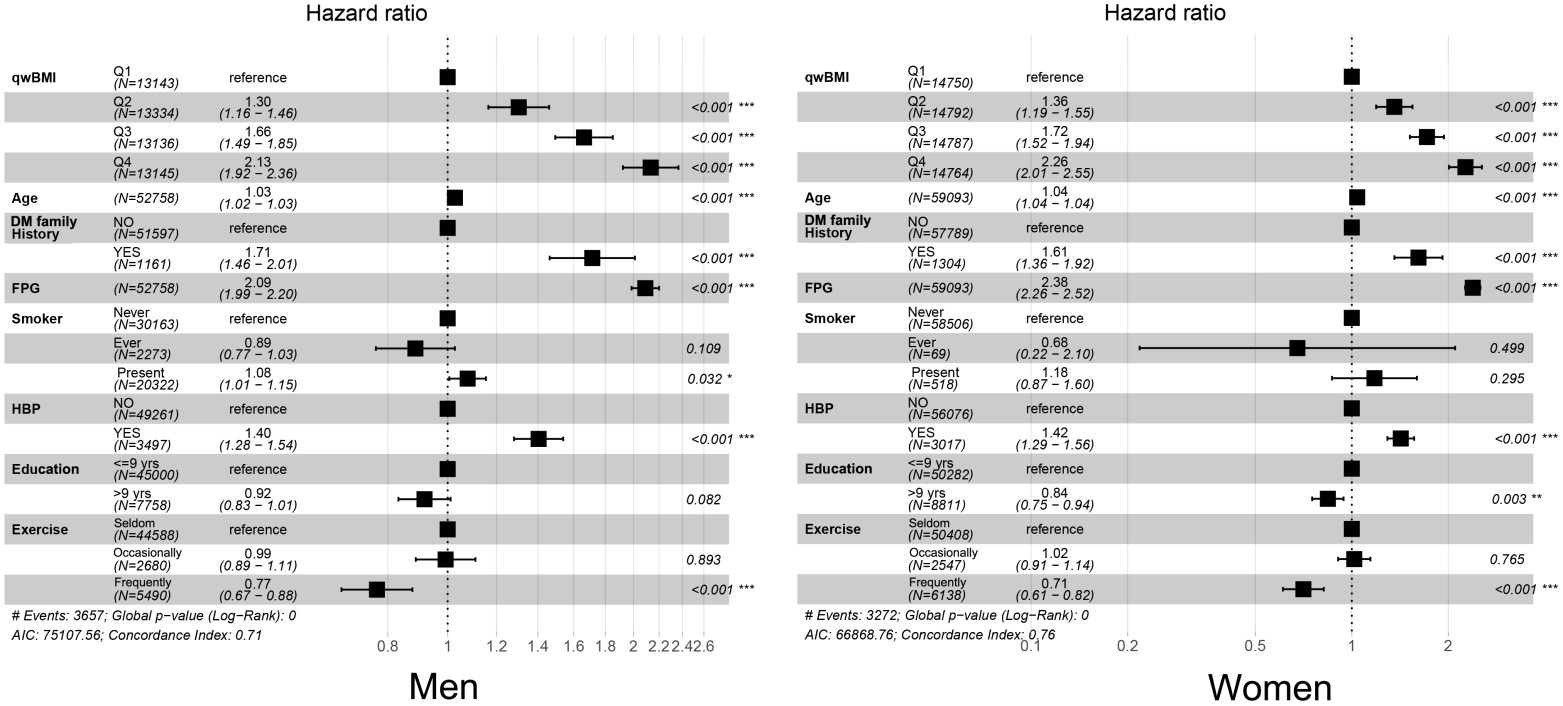
Forest plots of multivariable Cox regression results. After regression, quartiles of wBMI, age, DM family history, FPG, HBP status, education years, and exercise frequency were independent predictors for diabetes in men (left) and women (right). wBMI, waist-corrected body mass index; DM, diabetes mellitus; FPG, fasting plasma glucose; HBP, hypertension. *p< 0.05; **p< 0.01; ***p< 0.001.

After adjusting for multiple variables, WC, BMI, wBMI, and WHtR were still independent predictors for diabetes in both sexes, along with age, DM family history, FPG, HBP status, education years, and exercise habits. Notably, the adjusted HRs of wBMI for diabetes for the second, third, and fourth quartiles were 1.297 [95% CI: 1.157, 1.455], 1.664 [95% CI: 1.493, 1.853], and 2.132 [95% CI: 1.921, 2.366], respectively, when compared with the first quartile in men, and 1.357 [95% CI: 1.191, 1.546], 1.715 [95% CI: 1.517, 1.939], and 2.262 [95% CI: 2.010, 2.545] in women. Interestingly, HRs were higher in women than in men in models for WC, BMI, wBMI, and WHtR (**Table 2**, **Figure 2**).

### 3.3 wBMI is a better predictor of DM than other body composition measures

**Figure 3:**
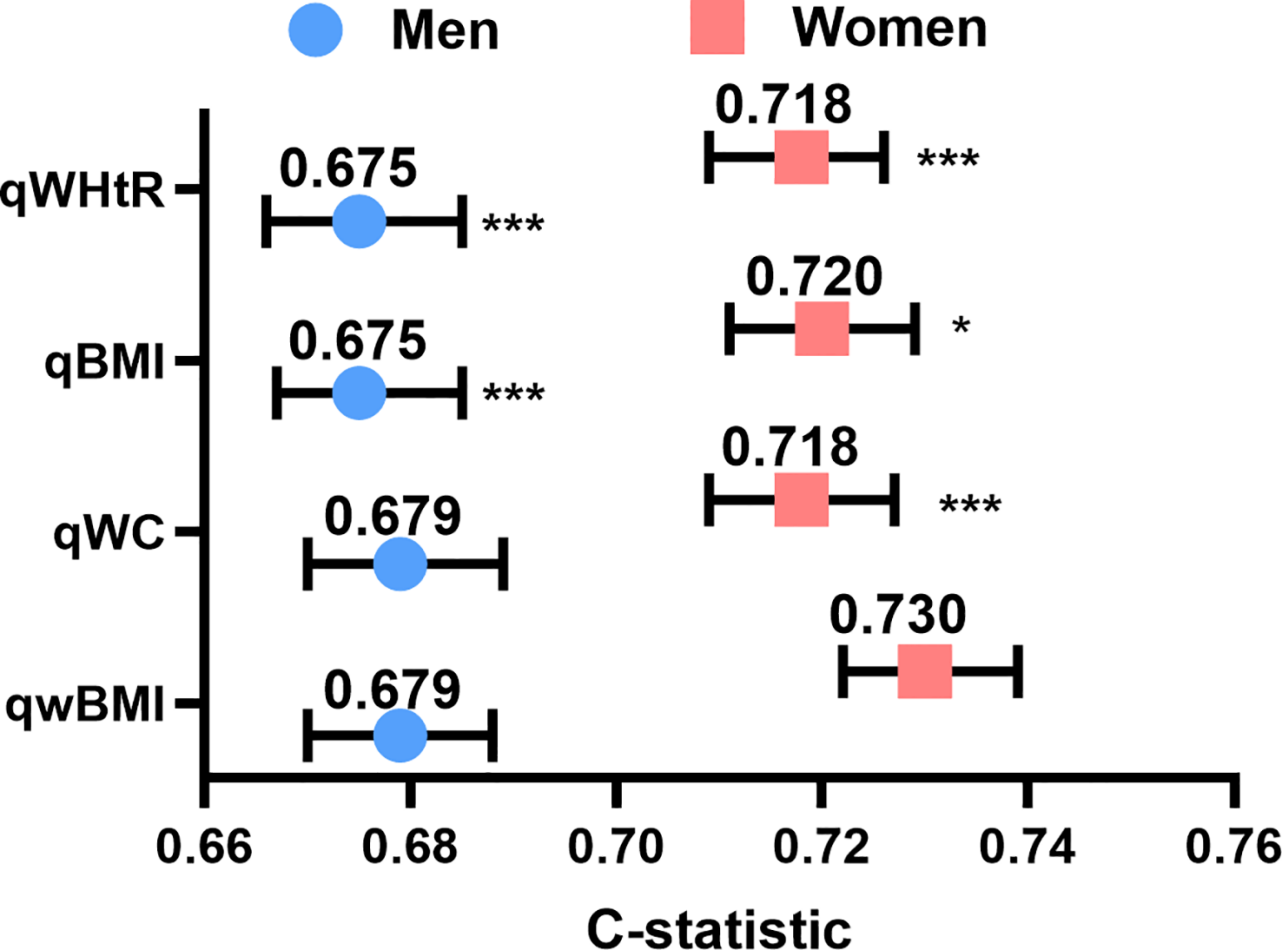
Concordance indexes of quartiles of wBMI, BMI, WC, and WHtR for both sexes. Compared to the other body composition measures, wBMI had the highest concordance in both men (left) and women (right). *p<0.05, ***p<0.001 *vs*. wBMI. qwBMI, quartile of waist-corrected body mass index; qWC, quartile of waist circumference; qBMI, quartile of body mass index; qWHtR, quartile of waist-to-height ratio.

To compare the predictive accuracy of variables, univariable Cox regression was used to calculate the C-index of WC, BMI, wBMI, and WHtR (**Figure 3**). wBMI had the highest C-index of all the predictors: 0.679 in men and 0.730 in women (men: 95% CI 0.670–0.688; women: 95% CI 0.722–0.739) (**Figure 3**). In men, wBMI was significantly different from BMI (p< 0.001) and WHtR (p< 0.001) but not from WC (p = 0.435). In women, wBMI was significantly different from WC (p = 0.0234), BMI (p< 0.001), and WHtR (p< 0.001). Notably, all C-indexes were higher in women than in men.

### 3.4 Nomogram of wBMI for incident DM

**Figure 4:**
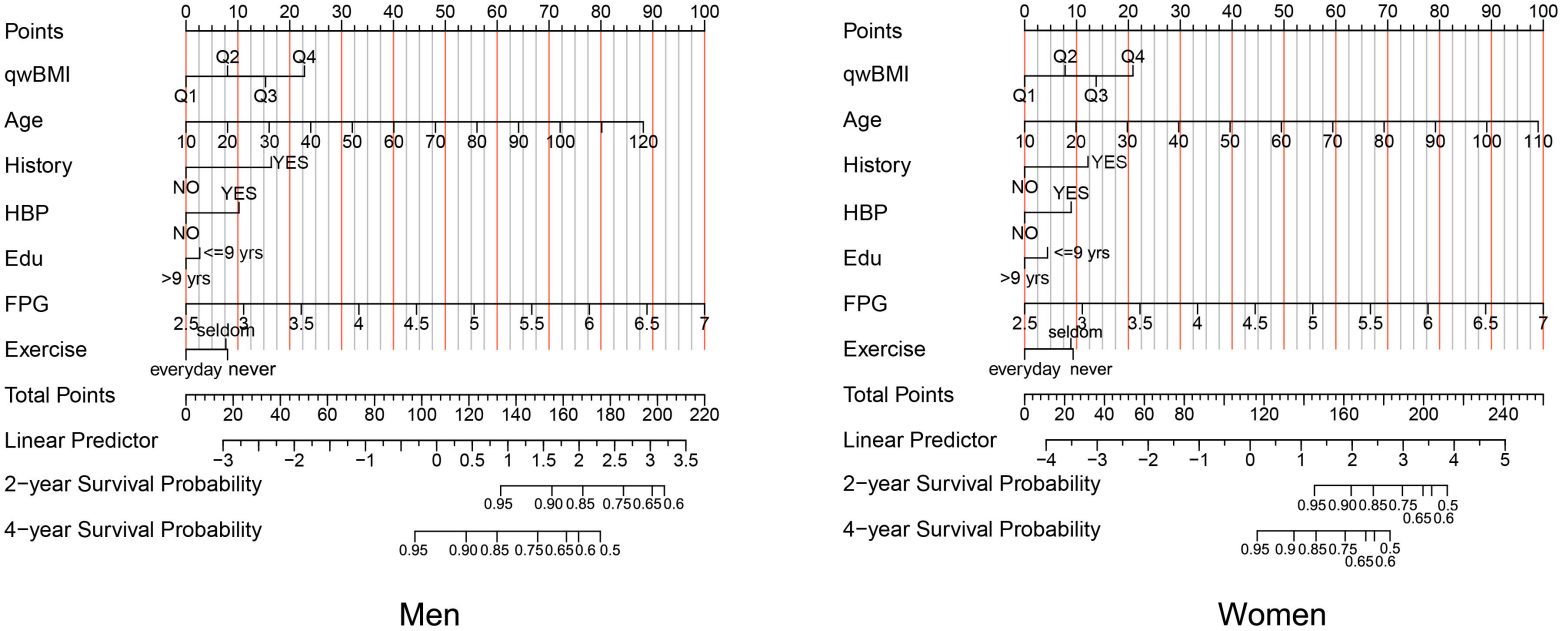
A nomogram for prognostic prediction of non-DM using qwBMI in men (left) and women (right). A male patient aged 60 years (40 points) with wBMI of 26.0 (15 points), no DM family history (0 points), a history of hypertension (10 points), education<9 years (3 points), FPG 6.0 mmol/L (78 points), and who seldom exercises (8 points) would have 154 total points. A line is drawn downward from the total points axis to the survival axes to determine probability of 2‐year non-DM (approximately 90%) and 4-year non-DM (approximately 70%). DM, diabetes mellitus; qwBMI, quartile of waist-corrected body mass index; FPG, fasting plasma glucose; HBP, hypertension.

We constructed a nomogram for incident DM in men and women that included the significant predictors identified by multivariable Cox analysis (**Figure 4**). Details of the individual prognostic scores of each risk factor are listed in **Supplementary Tables S2**; **S3**. The total nomogram score was determined based on the sum of individual scores. For example, a male patient with age 60 years (40 points), wBMI of 26.0 (15 points), no DM family history (0 points), history of hypertension (10 points), education<9 years (3 points), FPG of 6.0mmol/L (78 points), and who seldom exercises (8 points) would have a total score of 154 points. The subjects’ 2-year non-DM probability would be 90%, and the 4-year non-DM probability would be 70%.

**Figure 5:**
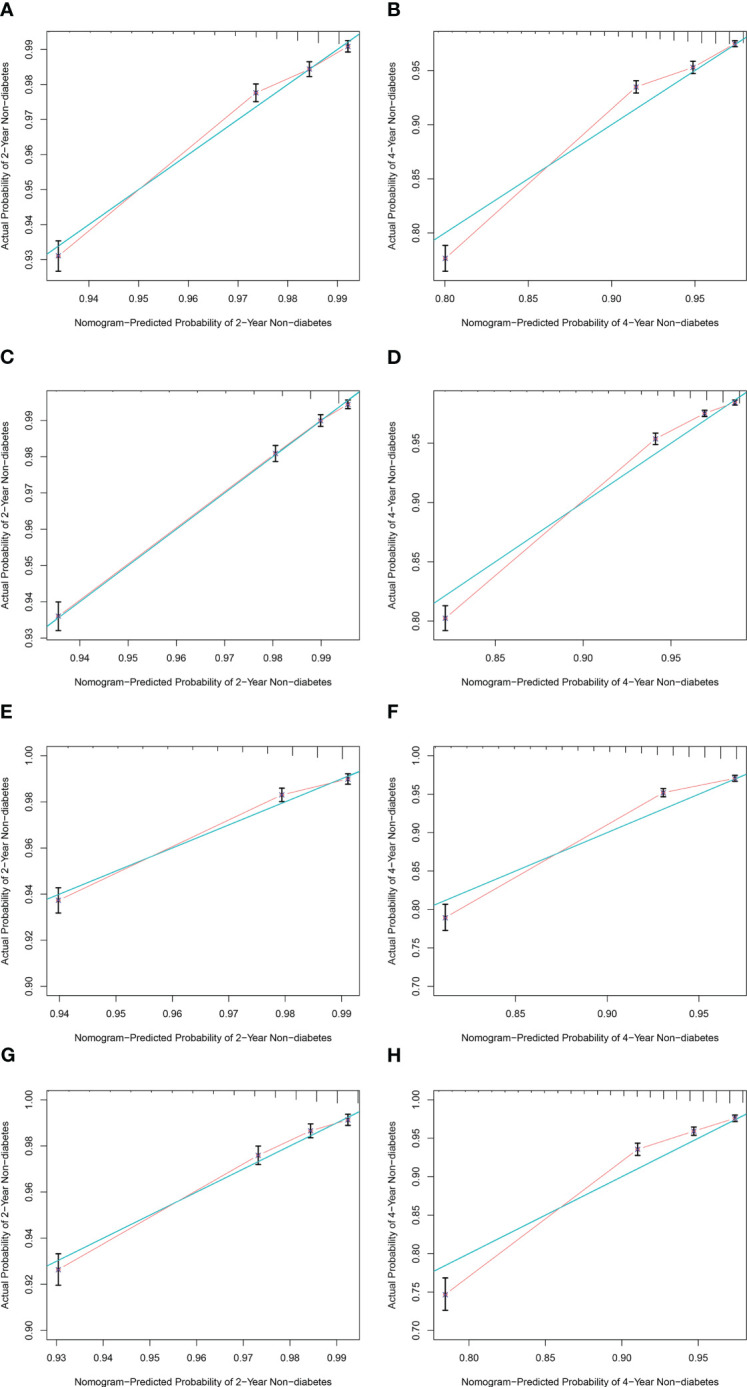
Calibration curves for the nomogram of both sexes. **(A, B)** Calibration curves of 2‐ and 4‐year non-DM probability for male subjects in the training cohort. **(C, D)** Calibration curves of 2‐ and 4‐year non-DM probability for female subjects in the training cohort. **(E, F)** Calibration curves of 2‐ and 4‐year non-DM probability for male subjects in the validation cohort. **(G, H)** Calibration curves of 2‐ and 4‐year non-DM probability for female subjects in the validation cohort. The green line indicates the ideal reference line where predicted probabilities would match observed survival rates. Red dots represent the performance of the nomogram and were calculated by bootstrapping. The closer the solid red line is to the green line, the more accurate the model’s predictions. DM, diabetes mellitus.

The C-index was 0.709 (95% CI: 0.700, 0.718, p< 0.001) for men and 0.759 (95% CI: 0.750, 0.767, p< 0.001) for women in the training cohort and 0.720 (95% CI: 0.707, 0.733, p< 0.001) for men and 0.738 (95% CI: 0.724, 0.752, p< 0.001) for women in the validation cohort, which indicates a medium discrimination ability of the model. A calibration plot was generated to assess the difference between nomogram-predicted and observed diabetes probability of the training and validation cohorts. The calibration curves showed high consistency between predicted and observed non-DM probabilities in both men and women when predicting 2- and 4-year non-diabetes probability both in the training (**Figures 5A–D**) and validation (**Figures 5E–H**) cohorts. In summary, the nomogram for DM showed acceptable discriminative and calibrating performance. We built an online calculator to predict incident DM probability based on our model, including variables of wBMI quartile, age, DM family history, FPG, hypertension history, education level, and physical exercise. The nomograms can be accessed at: https://huairen145.shinyapps.io/incident_DM_in_men/ for men and https://huairen145.shinyapps.io/DynNomapp/for women.

## 4 Discussion

In the present study of subjects in Tacheng Area in China, wBMI was a simple and important measure for predicting incident DM. The probability of developing DM increased for patients with wBMI in higher quartiles. When compared with C-indexes of WC, BMI, and WHtR, wBMI better predicted DM, especially for women. Finally, a nomogram was developed using wBMI and other important variables. This was the first large cohort study of the association of wBMI with incident DM.

BMI and WC are widely used and important clinical anthropometric parameters, especially for metabolic disease. Typically, BMI and WC are used separately to evaluate the impacts of body fat and shape on diabetes. The present findings showed that BMI, WC, and WHtR were all good predictors of incident DM, but a new indicator derived from the combination of BMI and WC (wBMI) was able to predict DM risk more effectively than other indexes, especially in women. BMI accounts for body fat mass but not distribution; WC and WHtR account for body fat distribution more than mass. The new index, wBMI, reflects both mass and distribution (17, 18), which may explain its advantage in predicting DM. Other studies have described the advantages of using both BMI and WC together, but did not use a unique indicator that combined them (20, 21).

In a previous study, wBMI, BMI, WC, and WHtR all showed good accuracy in identifying patients with insulin resistance, with wBMI having the largest area under curve (17). Insulin resistance is the mechanism underlying T2DM, so the strong association of wBMI with insulin resistance motivated us to further explore the relationship between wBMI and DM. Here, we demonstrated a definite prognostic function of wBMI for incident DM. wBMI and WC both outperformed BMI and WHtR when predicting DM in men, and wBMI was the strongest indicator in women. This sex difference was in accordance with a previous study (18).

Several limitations of this study need to be considered. DM was defined according to FPG, which is not as reliable as an oral glucose tolerance test. In addition, the current study only entailed a 4- to 5-year follow-up period. A follow-up study should be conducted to observe the longer-term occurrence of DM. Meanwhile, because of health checkup data limitation, we did not collect consumption of supplements that affected blood glucose (e.g., chromium). However, the large sample size and high follow-up rate in this study can reduce bias. Because this is the first investigation of predictive effects of wBMI on incident DM, it can serve as a reference for future studies.

In conclusion, incident DM risk increased with elevated quartiles of wBMI. wBMI had the strongest advantage for predicting DM when compared with WC, BMI, and WHtR, especially in women. A nomogram was developed according to wBMI and other variables identified as significant in multivariable regression analysis. This is the first large-sized cohort study on the association of wBMI with incident DM.

## Data availability statement

The original contributions presented in the study are included in the article/**Supplementary Material**. Further inquiries can be directed to the corresponding author.

## Ethics statement

The studies involving human participants were reviewed and approved by Ethical Review Committee of Shengjing Hospital of China Medical University. Written informed consent for participation was not required for this study in accordance with the national legislation and the institutional requirements.

## Author contributions

NW collected data, conceived and designed the experiments, analyzed data, and wrote the manuscript. YL collected data, conceived the experiment, and revised the manuscript. CG collected data, conceived and designed the experiments, and revised the manuscript. All authors contributed to the article and approved the submitted version.
