# Supplementary material for: Waist-corrected BMI predicts incident diabetes mellitus in a population-based observational cohort study
Source: Front Endocrinol (Lausanne). 2023 Jun 9;14:1186702. doi: 10.3389/fendo.2023.1186702 (PMC10290140; doi:10.3389/fendo.2023.1186702)
Supplement: Supplementary file 1 [file DataSheet_1.docx]

Check-up subjects (n=305,499)

Excluded n=127141

Baseline:

diabetes or hyperglycemia (n=26,532);

Missing wBMI (n=14,814);

Out of age (n=40,137);

Missing FPG (n=20,860);

Missing liver function, kidney function or lipid (n=21,190);

Severe liver dysfunction (n=722);

Severe kidney dysfunction (n= 759);

Malignancy (n=397);

Pregnancy (n=1,487)

Others, 243

Study population(n=178,358)

18,601 subjects excluded:

< 3 visits during study

47,906 in validation cohort

111,851 in training cohort

Figure S1. Flow chart of cohort selection.

Table S1. Baseline characteristics of participant by incident diabetes mellitus in males and females in validation cohort.

|  | Male | | | Female | | |
| --- | --- | --- | --- | --- | --- | --- |
|  | DM | NDM | P | DM | NDM | P |
| n | 1613 | 21017 |  | 1464 | 23812 |  |
| Age(yrs) | 52.0(12.6) | 45.2(13.7） | <0.001 | 54.2(12.4) | 45.2(13.2) | <0.001 |
| Pulse(rpm) | 76.6(18.5) | 75.3(25.0) | 0.024 | 76.5(21.2) | 76.2(26.5) | 0.036 |
| SBP(mmHg) | 127.6(10.3) | 125.6(10.4) | <0.001 | 123.7(11.4) | 121.3(11.2) | <0.001 |
| DBP(mmHg) | 80.2(9.1) | 78.3(8.1) | <0.001 | 75.5(9.2) | 74.2(7.4) | <0.001 |
| WC(cm) | 92.9(12.0) | 88.7(11.8) | <0.001 | 88.3(13.1) | 82.8(12.2) | <0.001 |
| BMI(kg/m2) | 26.9(4.0) | 25.5(3.8) | <0.001 | 26.5(4.5) | 24.6(4.0) | <0.001 |
| wBMI(kg/m2*m) | 25.3(6.6) | 22.9(5.9) | <0.001 | 23.7(7.0) | 20.7(6.0) | <0.001 |
| WHtR | 0.55(0.14) | 0.52(0.07) | <0.001 | 0.56(0.08) | 0.52(0.08) | <0.001 |
| DM family history (Yes, %(n)) | 4.6(74) | 2.1(432) | <0.001 | 4.1(50) | 2.1(504) | <0.001 |
| Urban (Yes, %(n)) | 38.8(626) | 38.7(8136) | 0.106 | 38.6(531) | 39.2(9464) | 0.008 |
| >9-year education (Yes, %(n)) | 12.6(203) | 15.0(3154) | 0.008 | 10.6(139) | 15.2(3621) | <0.001 |
| Exercise(%(n)) |  |  | <0.001 |  |  | <0.001 |
| Seldom | 80.5(1299) | 84.8(17822) |  | 83.2(1223) | 85.4(20320) |  |
| Occasionally | 12.5(201) | 4.7(978) |  | 10.5(147) | 3.9(1029) |  |
| Frequently | 7.0(113) | 10.5(2217) |  | 6.1(94) | 10.6(2463) |  |
| Diet(%(n)) |  |  | 0.100 |  |  | 0.047 |
| Mediterranean | 2.9(47) | 2.2(455) |  | 3.1(42) | 3.3(810) |  |
| Balance | 94.1(1518) | 94.4(19847) |  | 95.0(1398) | 94.2(22393) |  |
| Meat | 3.0(48) | 3.4(715) |  | 2.0(24) | 2.5(609) |  |
| Smoker(%(n)) |  |  | 0.007 |  |  | 0.379 |
| Never | 57.4(926) | 57.3(12052) |  | 98.6(1448) | 99.0(23600) |  |
| Ever | 5.7(92) | 4.1(867) |  | 0.09(3) | 0.1(19) |  |
| Present | 36.9(595) | 38.6(8098) |  | 1.3(13) | 0.9(193) |  |
| Drinker (%(n)) |  |  | 0.079 |  |  | 0.116 |
| Never | 59.4(958) | 62.0(13030) |  | 96.5(1420) | 95.8(22822) |  |
| Mild | 39.7(641) | 37.2(7815) |  | 3.2(44) | 4.0(985) |  |
| Heavy | 0.9(14) | 0.8(172) |  | 0.3(1) | 0.2(5) | Ref |
| HBP (Yes, %(n)) | 21.6(348) | 5.3(1110) | <0.001 | 21.6(301) | 4.1(1021) | <0.001 |
| CHD(Yes, %(n)) | 0.1(2) | 0.08(17) | 0.897 | 0.1(1) | 0.1(18) | 0.921 |
| Cerebral stroke (Yes, %(n)) | 0.1(2) | 0.1(24) | 0.911 | 0.3(2) | 0.2(52) | 0.714 |
| FPG(mmol/L) | 5.50(0.83) | 4.96(0.71) | <0.001 | 5.47(0.83) | 4.93(0.68) | <0.001 |
| ALT(U/L) | 29.7(21.6) | 27.5(19.0) | <0.001 | 23.3(17.0) | 20.7(14.7） | <0.001 |
| AST(U/L) | 24.4(13.4) | 24.0(11.9) | 0.249 | 22.7(11.5) | 21.7(10.9) | 0.002 |
| SCr(μmol/L) | 77.8(23.1) | 77.2(24.0) | 0.398 | 69.1(21.3) | 66.3(22.2) | <0.001 |
| TC(mmol/L) | 4.70(1.18) | 4.67(1.14) | 0.216 | 4.80(1.22) | 4.55(1.13) | <0.001 |
| TG(mmol/L) | 1.84(1.38) | 1.50(1.11) | <0.001 | 1.59(1.01) | 1.25 (0.89) | <0.001 |
| LDL(mmol/L) | 2.81(0.99) | 2.71(1.00) | 0.001 | 2.81(0.99) | 2.71(1.00) | 0.001 |
| HDL(mmol/L) | 1.40(0.60) | 1.46(0.61) | 0.002 | 1.49(0.56) | 1.54(0.57) | 0.004 |

Aberrations:

SBP, systolic blood pressure; DBP, diastolic blood pressure; WC, waist circumstance; BMI, body mass index; wBMI, waistcorrected BMI; WHtR, WaisttoHeight Ratio; DM, diabetes mellitus; HBP. Hypertension; CHD, coronary heart disease; FPG, fast plasma glucose; ALT, alanine aminotransferase; AST, aspartate aminotransferase; SCr, serum creatinine; TC, total cholesterol; TG, triglycerides; LDL, low density lipoprotein  ; HDL, high density lipoprotein.

Table S2. Table of risk score by risk factors in men.

|  | Risk Factor | Risk Score |
| --- | --- | --- |
| 1 | Age (years): |  |
|  | 10 | 0 |
|  | 20 | 8 |
|  | 30 | 16 |
|  | 40 | 24 |
|  | 50 | 32 |
|  | 60 | 40 |
|  | 70 | 48 |
|  | 80 | 56 |
|  | 90 | 64 |
|  | 100 | 72 |
|  | 110 | 80 |
|  | 120 | 88 |
| 2 | qwBMI |  |
|  | Q1 | 0 |
|  | Q2 | 8 |
|  | Q3 | 15 |
|  | Q4 | 23 |
| 3 | HBP |  |
|  | NO | 0 |
|  | YES | 10 |
| 4 | DM family history |  |
|  | NO | 0 |
|  | YES | 16 |
| 5 | Education |  |
|  | >9 yrs | 0 |
|  | <=9yrs | 3 |
| 6 | FPG |  |
|  | 2.8 | 0 |
|  | 3.0 | 11 |
|  | 3.5 | 22 |
|  | 4.0 | 33 |
|  | 4.5 | 44 |
|  | 5.0 | 56 |
|  | 5.5 | 67 |
|  | 6.0 | 78 |
|  | 6.5 | 89 |
|  | 7.0 | 100 |
| 7 | Exercise |  |
|  | Never | 8 |
|  | Seldom | 8 |
|  | Everyday | 0 |
|  | Total Risk Score: |  |

| Total Points 2-year Non-diabetes Probability | |
| --- | --- |
| 133 | 0.95 |
| 155 | 0.90 |
| 168 | 0.85 |
| 186 | 0.75 |
| 198 | 0.65 |
| 203 | 0.60 |

| Total Points 4-year Non-diabetes Probability | |
| --- | --- |
| 97 | 0.95 |
| 119 | 0.90 |
| 132 | 0.85 |
| 149 | 0.75 |
| 161 | 0.65 |
| 167 | 0.60 |
| 176 | 0.50 |

wBMI in men: Q1: ≤18.8; Q2: 18.9-22.3; Q3: 22.4-26.4; Q4: ≥26.5; In women: Q1: ≤16.7; Q2: 16.8-19.9; Q3: 20.0-23.9; Q4: ≥24.0

Table S3. Table of risk score by risk factors in women.

|  | Risk Factor | Risk Score |
| --- | --- | --- |
| 1 | Age (years): |  |
|  | 10 | 0 |
|  | 20 | 10 |
|  | 30 | 20 |
|  | 40 | 30 |
|  | 50 | 40 |
|  | 60 | 50 |
|  | 70 | 59 |
|  | 80 | 69 |
|  | 90 | 79 |
|  | 100 | 89 |
|  | 110 | 99 |
| 2 | qwBMI |  |
|  | Q1 | 0 |
|  | Q2 | 8 |
|  | Q3 | 14 |
|  | Q4 | 21 |
| 3 | HBP |  |
| NO | | 0 |
| YES | | 9 |
| 4 | DM family history |  |
| NO | | 0 |
| YES | | 12 |
| 5 | Education |  |
|  | >9 yrs | 0 |
|  | <=9yrs | 4 |
| 6 | FPG(mmol/L) |  |
|  | 2.8 | 0 |
|  | 3.0 | 11 |
|  | 3.5 | 22 |
|  | 4.0 | 33 |
|  | 4.5 | 44 |
|  | 5.0 | 56 |
|  | 5.5 | 67 |
|  | 6.0 | 78 |
|  | 6.5 | 89 |
|  | 7.0 | 100 |
|  | Exercise |  |
|  | Never | 9 |
|  | Seldom | 9 |
|  | Everyday | 0 |
|  | Total Risk Score: |  |

| Total Points 2-year Survival Probability | |
| --- | --- |
| 145 | 0.95 |
| 164 | 0.90 |
| 175 | 0.85 |
| 189 | 0.75 |
| 200 | 0.65 |
| 204 | 0.60 |
| 212 | 0.50 |

| Total Points 4-year Non-diabetes Probability | |
| --- | --- |
| 117 | 0.95 |
| 135 | 0.90 |
| 146 | 0.85 |
| 161 | 0.75 |
| 171 | 0.65 |
| 175 | 0.60 |
| 183 | 0.50 |
